# Preoperative recognition of appendiceal diverticulitis: a case report with multimodal correlation

**DOI:** 10.1093/jscr/rjag1007

**Published:** 2026-10-31

**Authors:** Nathan M Danh, Jun Guang Kendric Tan, Ruwan Wijesuriya

**Affiliations:** Department of General Surgery, St John of God Midland Public Hospital, 1 Clayton Street, Midland, Western Australia 6056, Australia; Department of General Surgery, St John of God Midland Public Hospital, 1 Clayton Street, Midland, Western Australia 6056, Australia; Department of General Surgery, St John of God Midland Public Hospital, 1 Clayton Street, Midland, Western Australia 6056, Australia

**Keywords:** appendiceal diverticulitis, appendiceal diverticulum, computed tomography, preoperative diagnosis, histopathology

## Abstract

Appendiceal diverticulitis is a rare clinicopathological entity that often mimics acute appendicitis and is infrequently recognized preoperatively. We report a 41-year-old woman presenting with migratory right iliac fossa pain in whom contrast-enhanced computed tomography (CT) prospectively identified a small inflamed appendiceal diverticulum with inflammatory change centered on it. Laparoscopic appendicectomy confirmed an inflamed distal appendiceal diverticulum. Macroscopic examination demonstrated a corresponding diverticulum, while histopathology confirmed an appendiceal pseudodiverticulum with acute diverticulitis and associated acute suppurative appendicitis. Review of recently published reports demonstrates variable multimodal documentation, with detailed radiological, intraoperative, macroscopic, and histopathological reporting across all four modalities remaining uncommon. This case provides complete multimodal correlation from prospective imaging diagnosis to histopathological confirmation and highlights the potential for accurate preoperative recognition when characteristic CT features are identified. Recognition may support operative management and definitive pathological assessment given reported associations with perforation and appendiceal neoplasia.

## Introduction

Appendiceal diverticula are rare outpouchings of the appendiceal wall, with a reported incidence of 0.004%–2.1% in appendicectomy specimens [1, 2]. They may be congenital true diverticula or, more commonly, acquired pseudodiverticula lacking muscularis propria [1, 2]. Inflammation of an appendiceal diverticulum, termed appendiceal diverticulitis, is a distinct clinicopathological entity from conventional acute appendicitis [1]. Its clinical presentation frequently mimics acute appendicitis, while reported associations with higher perforation rates and coexisting appendiceal neoplasms make recognition important [2–5].

Although characteristic computer tomography (CT) scan features have been described, appendiceal diverticulitis is infrequently recognized preoperatively, with most cases diagnosed only on histopathological examination for presumed acute appendicitis [2, 4, 6]. CT findings include an inflamed appendiceal diverticulum with focal inflammatory change centered on the diverticulum [4, 6]. We present a prospectively diagnosed case with detailed radiological, intraoperative, macroscopic and histopathological correlation.

## Case report

A 41-year-old woman presented with abdominal pain that had woken her overnight. The pain began in the periumbilical region before migrating to the right iliac fossa and radiating into the back. The pain was associated with nausea and subjective chills, but there was no vomiting or associated urinary or gynecological symptoms. Her medical history included hypothyroidism and two previous cesarean sections. She was hemodynamically stable and afebrile. Abdominal examination demonstrated focal tenderness and guarding in the right iliac fossa without generalized peritonism.

Laboratory investigations demonstrated a white cell count of 12.3 × 10^9^/l and C-reactive protein of 15.9 mg/l. Renal and liver function tests were normal, and serum β-human chorionic gonadotropin was negative. Contrast-enhanced CT demonstrated a prominent appendix with mild periappendiceal inflammatory stranding. A small appendiceal diverticulum was identified, with inflammatory change predominantly centered on the diverticulum and extending distally along the appendix (Fig. 1). No perforation, free intraperitoneal air, free fluid, or suspicious lymphadenopathy was identified. The radiological impression was acute uncomplicated appendicitis with associated appendiceal diverticulitis.

**Figure 1:**
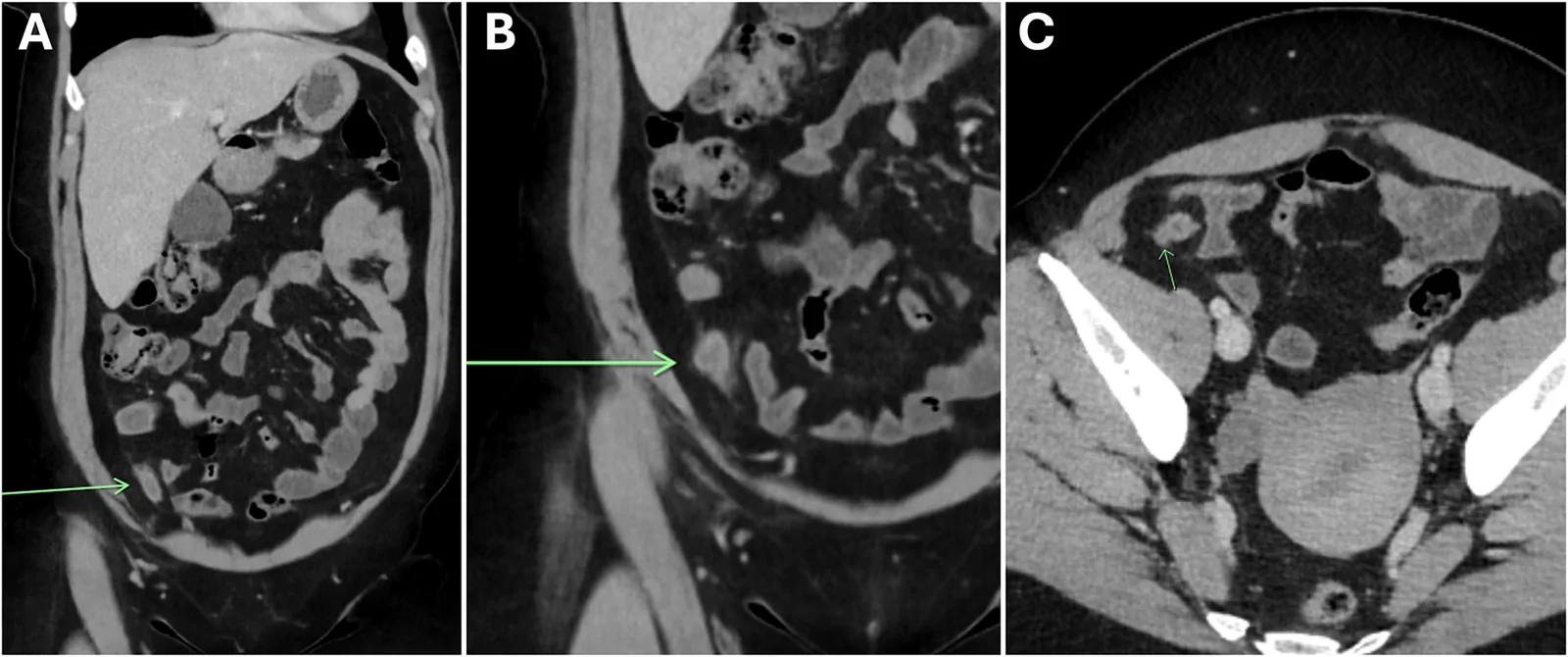
CT demonstrating prospective diagnosis of appendiceal diverticulitis. (A) Coronal CT image demonstrating inflammatory change involving the distal appendix (arrow). (B) Coronal CT image demonstrating a small inflamed appendiceal diverticulum with surrounding periappendiceal inflammatory change (arrow). (C) Axial CT image confirming the inflamed appendiceal diverticulum (arrow).

Laparoscopic appendicectomy was performed the following day. An inflamed appendix with a diverticulum arising from its distal aspect was identified (Fig. 2), without perforation, abscess or generalized contamination. Recovery was uncomplicated, and the patient was discharged on postoperative day one. Macroscopic examination demonstrated an 8 × 6 mm cream-gray nodular lesion at the distal appendix (Fig. 3). Histopathological examination demonstrated an appendiceal pseudodiverticulum lacking muscularis propria, with marked transmural acute inflammatory cell infiltration extending to the serosa and associated acute serositis, consistent with acute diverticulitis (Fig. 4). The appendix also demonstrated extensive mucosal ulceration lined by acute inflammatory exudate, transmural neutrophilic inflammation and focal necrosis, consistent with acute suppurative appendicitis. No granulomatous inflammation, dysplasia or malignancy was identified. At outpatient follow-up, the patient had recovered without complications and was discharged to the care of her general practitioner.

**Figure 2:**
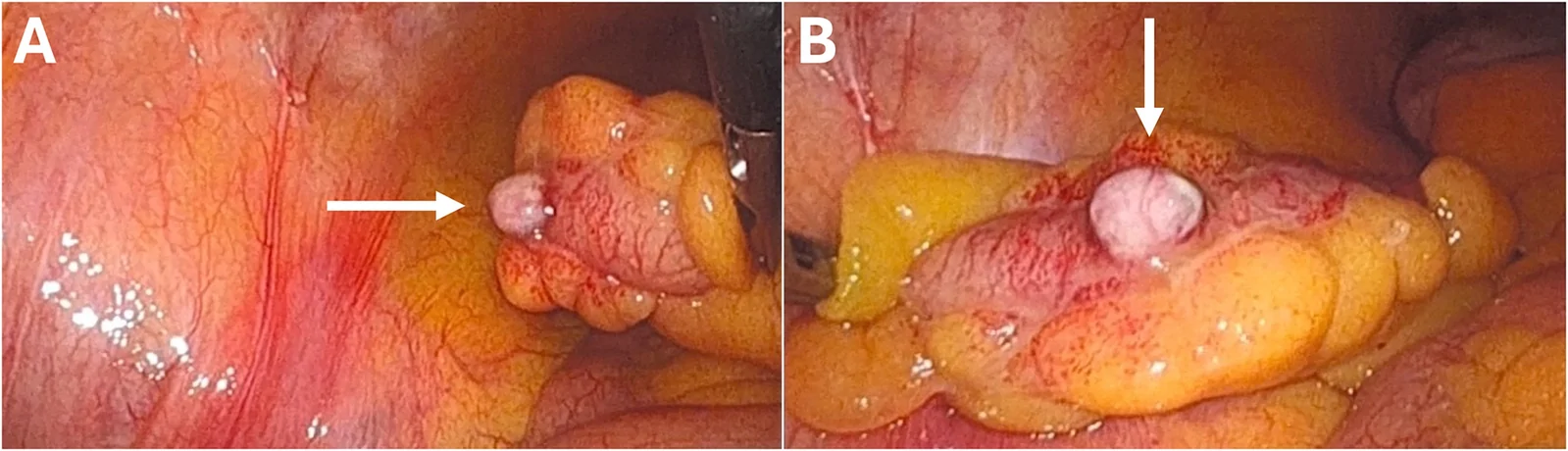
Intraoperative identification of appendiceal diverticulitis. (A) Laparoscopic image demonstrating an inflamed appendiceal diverticulum arising from the distal appendix (arrow). (B) Close-up laparoscopic image demonstrating the inflamed appendiceal diverticulum (arrow).

**Figure 3:**
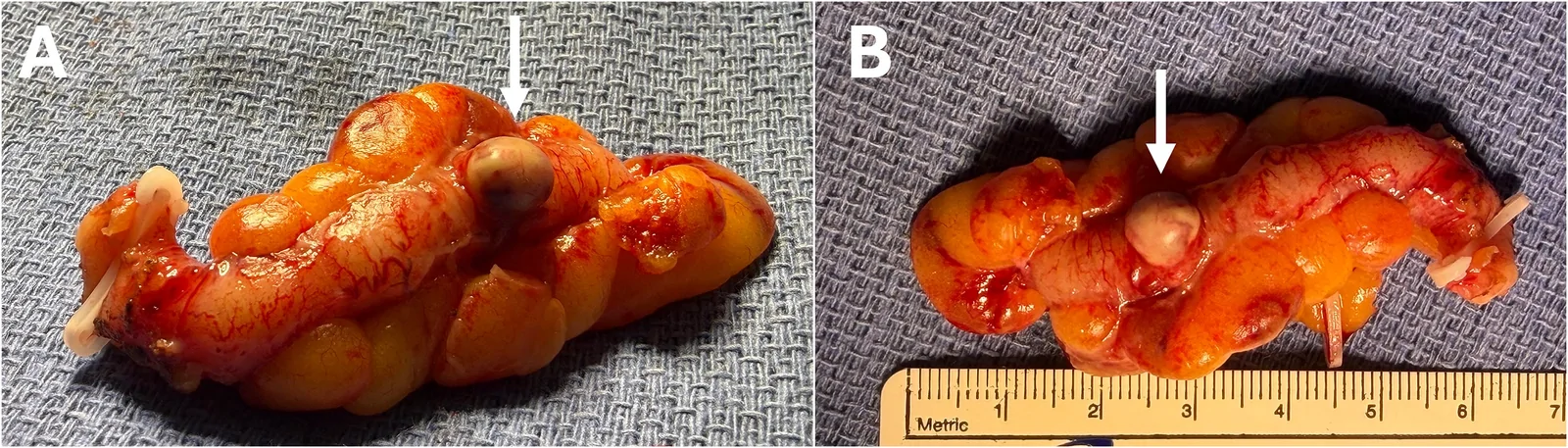
Gross pathological appearance of appendiceal diverticulitis. (A) Gross specimen demonstrating an inflamed appendiceal diverticulum arising from the appendiceal wall (arrow). (B) Gross specimen with adjacent ruler demonstrating the appendiceal diverticulum (arrow) and specimen size.

**Figure 4:**
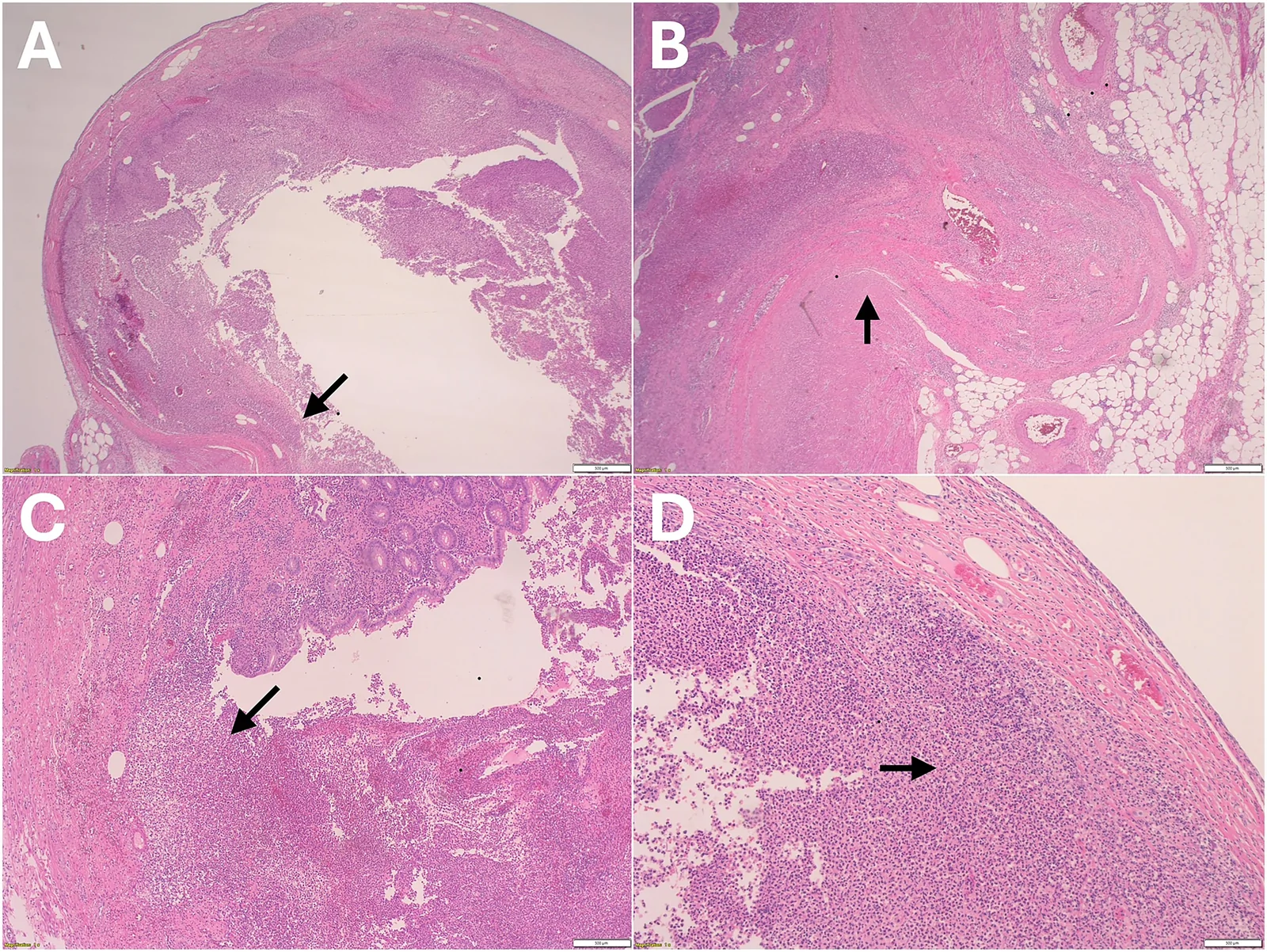
Histopathological findings of appendiceal diverticulitis (hematoxylin and eosin staining). (A–B) Low-power H&E-stained sections demonstrate an appendiceal pseudodiverticulum (arrows). The wall of the pseudodiverticulum is formed by the submucosa, subserosa, and serosa, lacking the muscularis propria. The diverticulum demonstrates marked transmural acute inflammatory cell infiltration with overlying acute serositis**.** (C) High-power H&E-stained section shows the appendiceal lumen with extensive areas of mucosal ulceration. The ulcerated mucosal surface is lined by acute inflammatory exudate. Transmural neutrophilic infiltration is present with overlying acute serositis. The black arrow highlights the acute inflammatory exudate. (D) High-power H&E-stained section of the pseudodiverticulum showing acute inflammatory exudate filling the diverticular lumen and extending to the subserosa. The black arrow highlights the acute inflammatory exudate extending to the subserosa of the pseudodiverticulum. Scale bars are shown in each panel.

## Discussion

Appendiceal diverticulitis is increasingly recognized as a distinct clinicopathological entity from conventional acute appendicitis, with differences in its pathological characteristics, clinical behavior, radiological findings, and associated complications. Although uncommon, recognition is important because of its reported associations with higher perforation rates and coexisting appendiceal neoplasms, and because it remains difficult to distinguish from acute appendicitis preoperatively [2, 3, 5, 6]. Published series indicate that appendiceal diverticulitis predominantly affects adults, often at an older age than conventional acute appendicitis, and has a male predominance [2, 3, 6]. Although our patient was female, her age of 41 years is consistent with the reported age distribution.

The clinical importance of appendiceal diverticulitis is underscored by its substantially greater propensity for perforation compared with conventional acute appendicitis. Ito *et al.* reported perforation rates of 68% and 27% (*P* < .001), respectively, while Yardimci *et al.* reported rates of 70% and 26% [3, 4]. Sahin *et al.* identified intraoperative perforation in 32.7% of patients with appendiceal diverticular disease and histological evidence of diverticular perforation in 40.5%, suggesting that perforation may be under-recognized intraoperatively [2]. This susceptibility is thought to reflect the structural weakness of acquired pseudodiverticula, which lack muscularis propria [1, 3].

Appendiceal diverticular disease is also associated with appendiceal neoplasms, including neuroendocrine tumors, low-grade appendiceal mucinous neoplasms and adenocarcinomas, although reported prevalence varies considerably between published series [1, 2, 5, 6]. Current evidence supports coexistence of appendiceal diverticular disease and appendiceal neoplasia, rather than malignant transformation arising from the diverticulum itself [2]. Careful macroscopic and histopathological assessment of appendicectomy specimens is therefore important to exclude neoplasia [1, 5, 6].

Preoperative diagnosis remains challenging because the clinical presentation usually resembles acute appendicitis and imaging findings may be subtle [1, 3, 4, 6]. In a systematic review, right iliac fossa or right lower quadrant pain was reported in 56.5% of patients, with nausea and vomiting in 18.9% and 9.8%, respectively [6]. CT remains the imaging modality of choice, with characteristic features including an inflamed appendiceal diverticulum, peri-diverticular inflammatory change, periappendiceal fat stranding or fluid, and inflammation centered on the diverticulum rather than the appendix [3, 4, 6]. In the series by Sahin *et al.,* none of 153 patients were diagnosed on the initial CT report, although retrospective review identified appendiceal diverticula in 59.1% of available examinations [2].

The present case demonstrates the potential for prospective diagnosis of appendiceal diverticulitis when characteristic CT findings are recognized. CT prospectively identified a small appendiceal diverticulum with inflammation centered on it, allowing prospective diagnosis of appendiceal diverticulitis (Fig. 1). This was confirmed intraoperatively and correlated with macroscopic and histopathological findings (Figs 2–4). Review of ten recently published reports demonstrated considerable variability in multimodal documentation, with detailed radiological, intraoperative, macroscopic, and histopathological reporting remaining limited (Table 1) [6–15]. Several reports combined radiological, intraoperative, macroscopic and/or histopathological findings, although detailed reporting across all four modalities remained uncommon. The present case adds to this limited literature by providing complete correlation across all four modalities, illustrating that appendiceal diverticulitis can be recognized prospectively when its characteristic CT features are identified. Preoperative recognition may have important management implications: the reported risks of perforation and coexisting neoplasia may support appendicectomy rather than non-operative management, while also providing tissue for definitive histopathological assessment [1–6].

**Table 1:** Comparison of the diagnostic pathway and clinicopathological documentation among recently published illustrative case reports of appendiceal diverticulitis and the present case

**First author**	**Year**	**Age/sex**	**Prospective CT diagnosis**	**Intraoperative image**	**Gross specimen image**	**Histopathology image**	**Perforation**	**Associated neoplasm**
Abdelrahim *et al.*	2024	50s M	Yes	Yes	Yes	No	No	No
Böttger *et al.*	2024	55 M	No	No	Yes	Yes	Yes	No
Saidi *et al.*	2025	34 M	Yes	Yes	Yes	No	No	No
Chen *et al.*	2025	58 M	Yes	Yes	No	Yes	No	No
Sousa *et al.*	2025	37 F	No	No	No	Yes	Yes	No
Furey *et al.*	2025	61 F	No	No	No	Yes	No	No
Gandhi *et al.*	2025	50 M	No	No	Yes	Yes	No	No
Vidović *et al.*	2025	53 M	No	Yes	Yes	Yes	No	No
Ababneh *et al.*	2026	34 F	No	No	Yes	Yes	Yes	No
Ben Ismail *et al.*	2026	27 M	No	Yes	Yes	No	No	No
Present case	2026	41 F	Yes	Yes	Yes	Yes	No	No
